# Erection of an Edifice for the Buffalo Medical College

**Published:** 1847-09

**Authors:** 


*Erection of an Edifice for the Buffalo Medical College.*—We are gratified in being able to announce to our readers, that a sufficient sum has been subscribed by the citizens of this place, to secure the erection of a commodious and ornamental edifice for the Medical Department of the University of Buffalo. With the conviction that a suitable structure is alone necessary to place the school on a permanent basis, and secure its prosperity, the friends of the institution have just reason to congratulate themselves on the liberality and public spirit which have so promptly and efficiently responded to their appeal. The building at present occupied by the College is leased for a short period, and although it answers exceedingly well for the present, is not adapted to meet the prospective wants of the institution. The new edifice will probably be erected during the next summer.

